# Initiation of statin therapy halts progression of atherosclerotic plaque burden in peripheral arterial disease

**DOI:** 10.1186/1532-429X-11-S1-O19

**Published:** 2009-01-28

**Authors:** Amy M West, Justin D Anderson, Craig D Meyer, Frederick H Epstein, Klaus D Hagspiel, Stuart S Berr, Nancy L Harthun, Joseph M DiMaria, Jennifer R Hunter, John M Christopher, Gabriel B Winberry, Christopher M Kramer

**Affiliations:** University of Virginia, Charlottesville, VA USA

**Keywords:** Simvastatin, Statin Therapy, Peripheral Arterial Disease, Ezetimibe, Lipid Lowering Therapy

## Introduction

Studies suggest lipid lowering therapy improves symptoms and exercise performance in patients with peripheral arterial disease (PAD); however, the mechanism of action is unclear.

## Purpose

We sought to use CMR to study the relationship between LDL reduction and superficial femoral artery (SFA) plaque burden in patients with PAD treated with lipid lowering therapy over the course of 1 year.

## Methods

63 patients with mild-to-moderate symptomatic PAD (mean age 63 ± 10 years, mean ankle brachial index (ABI) 0.69 ± 0.15) had their most symptomatic leg studied with MRI to assess atherosclerotic plaque burden before and 1 year after being started on lipid lowering therapy. At study entry, statin-naïve patients were randomized to either simvastatin 40 mg or simvastatin 40 mg plus ezetimibe 10 mg (R group, n = 31) while patients already on a statin were given open-label ezetimibe 10 mg (Z group, n = 32). Lipid measurements were obtained as part of the VAP test. CMR was performed using fat-suppressed multi-slice turbo-spin-echo pulse sequence on a Siemens Avanto 1.5 T scanner. A custom-built flexible, linear four-element (10 cm × 10 cm square element) surface coil array was placed over the SFA to image 15–20 cm along the vessel beginning below the bifurcation of the common femoral. Blood was suppressed through the multislice data set using spatial presaturation, with periodic excitation of upstream slices. Imaging parameters included: repetition time 1100 ms, echo time 7.6 ms, echo spacing 7.5 ms, turbo factor (9), voxel size 0.5 × 0.5 × 3 mm, 4 signal averages, with interleaved image sets. Plaque volume (PV) defined as total vessel volume (TVV) minus lumen volume (LV) was measured with VesselMass software. Changes in all parameters between groups from baseline to year one were compared by unpaired t-test. Changes in LDL were compared with changes in plaque parameters by linear regression.

## Results

**Figure 1:**
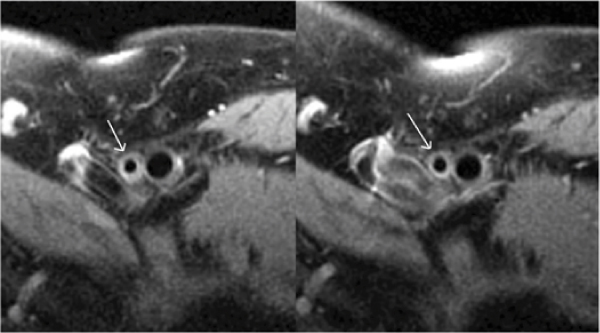
**Left – Representative black blood image of SFA from R group patient at baseline**. Right – same location imaged one year later. Notice plaque regression in the SFA.

LDL at baseline was higher in the R group (120 ± 37) than the Z group (100 ± 27) mg/dl, p = 0.02. The decrease in LDL at one year was significantly greater in R (-41 ± 37) than Z (-21 ± 30) mg/dl, p < 0.03, such that final LDL was similar between groups (79 ± 36 in the R group and 79 ± 33 in the Z group, mg/dl). The total cholesterol at baseline was higher in the R group (195 ± 42) than the Z group (171 ± 39) mg/dl, p < 0.03. There was a trend towards a greater fall in total cholesterol in R (48 ± 42) compared to Z (27 ± 42) mg/dl, p = 0.07. The final total cholesterol was similar between groups. No between group changes in HDL or triglycerides were seen. See Table 1 for changes in vessel wall parameters in the 2 groups. Plaque volume regressed in R while it progressed in Z and total vessel volume followed the same trend (p = 0.11). No between group differences in lumen volume or change in lumen volume over time was noted. No correlation was found between change in LDL and plaque volume in the SFA. Figure 1.

**Table 1:** Changes in plaque and vessel wall volume over time

	Baseline	One Year	% Change
Plaque volume (cm3) – R	8.64 ± 4.72	8.50 ± 4.70	-2 ± 8%*
Plaque volume (cm3) – Z	9.16 ± 4.94	9.53 ± 4.82	+3 ± 11%
TVV (cm3) – R	13.61 ± 7.99	13.34 ± 7.93	-2 ± 8%
TVV (cm3) – Z	14.27 ± 6.88	14.64 ± 6.83	+1 ± 8%

*p < 0.04 vs. Z group

## Conclusion

Statin-naïve patients with PAD who were begun on either simvastatin or simvastatin plus ezetimibe for one year had a halting of progression of atherosclerotic plaque volume in the superficial femoral artery when compared to those already treated with statin given ezetimibe. Reverse vessel wall remodeling was noted among PAD patients newly treated with statins compared to those with ezetimibe added to pre-existing statin therapy. Thus the degree and/or mechanism of LDL lowering rather than the final LDL achieved may be important in halting atherosclerotic plaque progression. In addition, this study demonstrates that CMR can show differences in plaque progression in PAD with small numbers per patient group.

